# The tube-plugging test: a simple assay that reveals offspring-centered defensive behavior in postpartum mice

**DOI:** 10.3389/fnbeh.2026.1767805

**Published:** 2026-05-26

**Authors:** Noriko Horii-Hayashi, Shoma Miki, Hanano Takashima, Koshiro Akaki, Koichi Inoue

**Affiliations:** Department of Anatomy and Cell Biology, Nara Medical University, Kashihara, Nara, Japan

**Keywords:** defensive behavior, hypothalamus, lactation, maternal anxiety, maternal behavior, offspring-centered defense, perinatal mental health, postpartum

## Abstract

Perinatal mental health is a major public health concern. Epidemiological studies indicate that approximately 10–20% of women in the postpartum period experience clinically significant depressive or obsessive-compulsive symptoms. Many postpartum symptoms involve heightened vigilance and behaviors aimed at protecting the infant from possible harm, suggesting the presence of offspring-centered defensive processes. One offspring-centered defensive behavior described in wild rats is entrance-sealing, in which lactating females plug the entrance of their burrow to limit access by potential intruders. Although laboratory mice rarely exhibit such behavior spontaneously, similar bedding-plugging behavior has been occasionally observed, suggesting that mice retain the capacity for this response. However, no method has existed for reliably quantifying such behavior under controlled laboratory conditions. To address this gap, we developed the Tube-Plugging Test (TPT), an assay that measures bedding accumulated at tube-like openings attached to the home cage and enables repeated, non-invasive quantification of plugging behavior. Using this approach, we found that tube plugging occurred rarely in males, intermittently in virgin females, and most robustly in postpartum females. Notably, plugging behavior exhibited substantial inter-individual variability, but was more temporally stable across days in postpartum females than in virgin females. In postpartum females, exposure to a male intruder altered plugging behavior at the tube through which the intruder was introduced, suggesting that plugging is modulated by direct social context. Together, these findings establish the TPT as a simple and reproducible method for quantifying plugging behavior and identify tube plugging as a measurable component of offspring-centered defensive behavior.

## 1 Introduction

**Figure 1:**
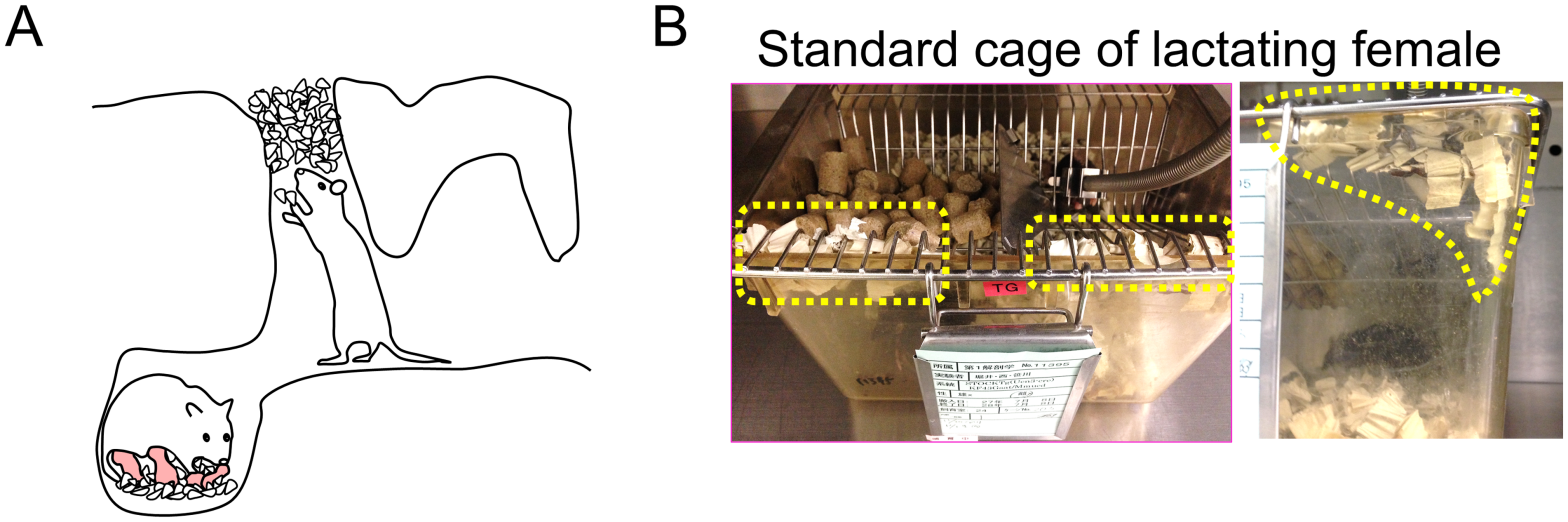
Maternal entrance-sealing behavior. **(A)** A schematic illustration of entrance-sealing behavior originally described in wild rats. The illustration was adapted from Horii-Hayashi et al. (2024). Lactating females plug the entrance of their burrow with mud, soil, or vegetation to block access by potential intruders while pups remain inside the nest. **(B)** Examples of bedding accumulated in gaps of the cage lid (left, dotted line) and in an upper corner of the home cage (right, dotted line) in lactating females.

Motherhood is accompanied by neurobiological adaptations anchored in hormonal changes and specific hypothalamic–limbic circuits that increase maternal motivation and vigilance toward offspring cues (Feldman, 2015, 2016). In rodents, these adaptations support a broad repertoire of maternal behaviors, including lactation, nest building, pup retrieval, and maternal aggression in response to immediate and overt threats such as intruders (Feldman, 2016). While maternal aggression has been extensively studied as a defensive response to direct and immediate threats (Lonstein and Gammie, 2002; Puska et al., 2024), defensive strategies that modify the surrounding environment in the absence of direct confrontation have received comparatively less attention. In natural environments, mothers often modify their surroundings to protect their offspring. For example, in wild rats, lactating females block the burrow entrance to limit intrusion (Calhoun, 1963) (Figure 1A). Similarly, in Mexican cottontail rabbits (*Sylvilagus cunicularius*), females nurse their young at the burrow entrance and close the entrance after each visit during early development, effectively restricting access to the nest (Rodríguez-Martínez et al., 2014). These entrance-sealing behaviors are thought to protect offspring from predators or intruders that have not yet arrived, representing a defensive strategy against potential threats. Together, these observations suggest that such behaviors may be a component of maternal defense across mammalian species.

Under standard laboratory housing conditions, opportunities to observe entrance-sealing behavior in mice are limited. More broadly, commonly used laboratory behavioral assays provide limited opportunities to examine defensive behaviors that are specifically oriented toward offspring protection. Nevertheless, lactating mice occasionally pack bedding material or feces into gaps beneath the cage lid (Figure 1B), indicating that entrance-sealing–like behavior can occur even under standard laboratory conditions. These observations suggest that laboratory mice retain the capacity for this type of behavior. However, a systematic and quantitative method to assess this behavior in laboratory mice has been lacking. Quantitative assessment of entrance-sealing behavior will be important for understanding how the maternal brain adapts to protect offspring from potential threats.

To address this gap, we developed the Tube-Plugging Test (TPT), a simple and quantifiable paradigm that enables repeated measurement of entrance-sealing behavior under standard housing conditions. Using this approach, we examined how sex and reproductive state influence entrance-sealing behavior and whether sealing in lactating females is modulated by exposure to an intruder. Together, these experiments establish the TPT as a practical tool for investigating environmental modification aimed at protecting offspring from potential threats.

## 2 Materials and methods

### 2.1 Animals

Adult C57BL/6N and ICR mice (8–24 weeks old) of both sexes were used. C57BL/6N mice served as the main experimental strain, whereas ICR mice were included for supplementary strain comparison experiments (Supplementary Figure S1). Both strains were purchased from CLEA Japan (Tokyo, Japan). Mice were maintained in a temperature- and humidity-controlled facility (25 °C; 40–60% humidity) under a 12:12 h light/dark cycle (lights on at 08:00). Food and water were available ad libitum. Standard paper-based bedding was used.

Prior to pregnancy, all mice were group-housed (3–5 per cage) under standard laboratory conditions to minimize potential stress associated with single housing. Once pregnancy was visually confirmed by abdominal enlargement, females were single-housed and remained individually housed throughout parturition and lactation. Female mice were tested either as virgins or as postpartum mothers, and postpartum females were tested during the early postpartum period. Litter size was not experimentally standardized; postpartum females naturally had litters of 5–8 pups. Females that exhibited infanticide were excluded from the study. All procedures were approved by the Institutional Animal Care and Use Committee of Nara Medical University and were conducted in accordance with national guidelines for the care and use of laboratory animals.

### 2.2 TPT apparatus

**Figure 2:**
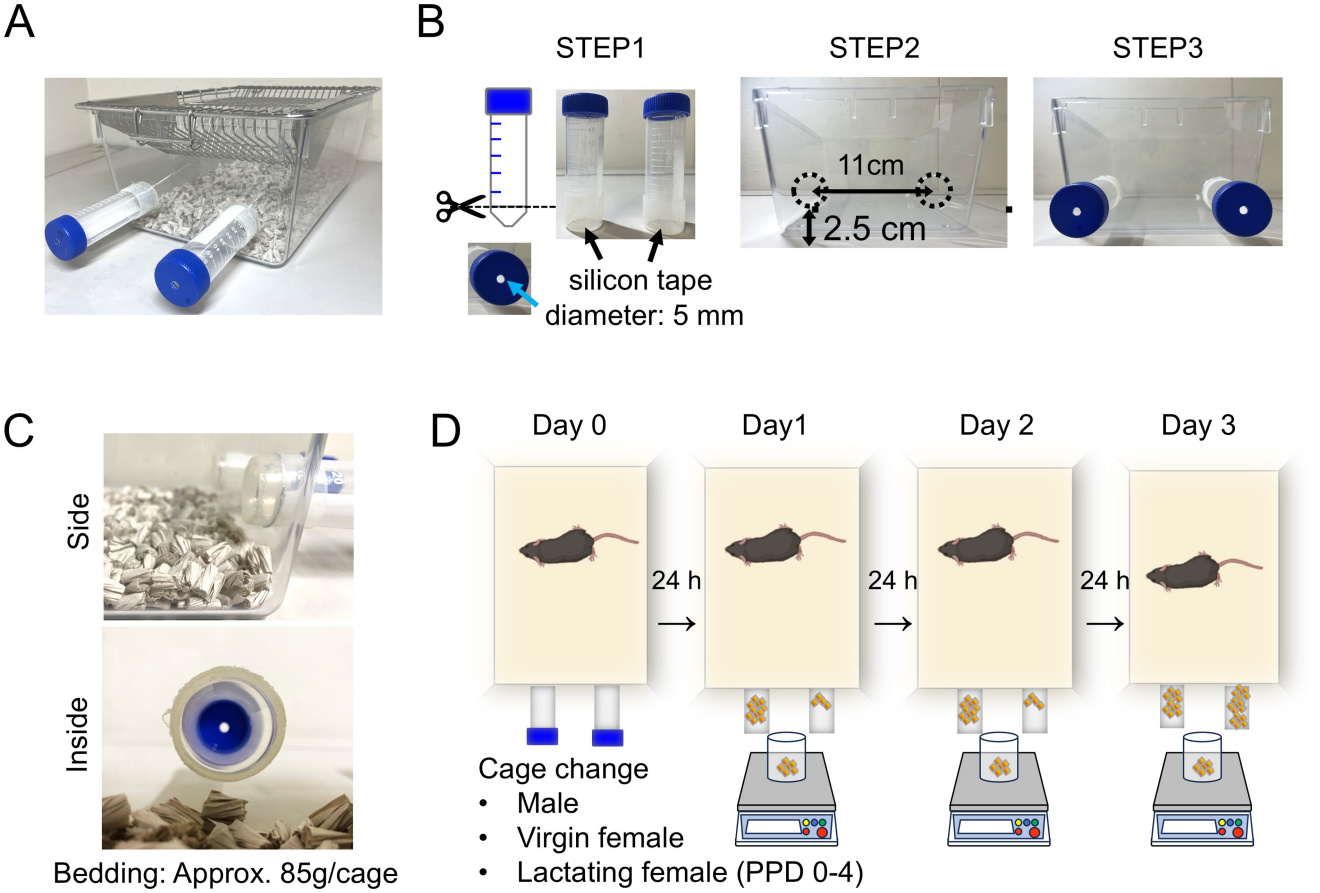
Apparatus and procedure for the TPT. **(A)** Photograph of the TPT cage used in this study. **(B)** Construction of the TPT apparatus: STEP 1: The conical bottom of a 50-mL plastic centrifuge tube was removed, and the cut edge was wrapped with silicone tape. A 5-mm-diameter hole was made in the tube cap. STEP 2: Two circular holes (11 cm apart) were drilled in the side wall of a standard mouse cage. STEP 3: The modified tubes were inserted through the holes, with the capped ends positioned outside the cage. **(C)** Representative images showing the relationship between bedding and the tube openings. Upper panel: side view of the tube inserted into the cage wall with bedding material. Lower panel: inside view of the tube opening. Approximately 85 g of bedding was provided per cage. **(D)** Experimental procedure for the TPT. Mice (males, virgin females, or postpartum females) were transferred individually to the TPT cage on Day 0. For postpartum females, transfer occurred between postpartum day (PPD) 0 and 4. After each 24-h session (Day 1–Day 3), the outer caps were removed and bedding plugged inside the tube openings was collected and weighed to quantify plugging intensity. This schedule ensured that measurements in postpartum females were completed within the early postpartum period (PPD3–7).

The TPT apparatus consisted of a standard mouse home cage fitted with two modified 50-mL plastic centrifuge tubes serving as access tubes (Figure 2A). Each tube was prepared by cutting off the conical bottom of a 50-mL tube (Falcon, Corning Inc., NY, USA) and wrapping the open end with silicone laboratory tape (Figure 2B: step 1). A small hole (5 mm in diameter) was made in the center of each cap (Figure 2B: step 1). A circular hole was drilled on each side of the cage wall (11 cm apart) (Figure 2B, step 2) and the modified tubes were inserted through these holes such that the capped ends remained outside the cage while the open ends faced the inside of the cage (Figure 2B, step 3).

The tubes were primarily secured by friction between the taped tube surface and the cage wall. When necessary, additional tape was applied externally to maintain stable positioning during testing. The inner opening of each tube served as the site where bedding could be packed by the mouse. All sealing measurements were performed using the same pair of tubes across sessions.

Approximately 85 g of bedding material was provided per cage to ensure that bedding availability did not limit behaviors such as nest building. The bedding level was maintained below the height of the tube opening to minimize accidental entry into the tube (Figure 2C).

### 2.3 TPT procedure

All mice, including males, virgin females, and postpartum females, were transferred to an individual testing cage equipped with the tube apparatus 24 h before the first plugging measurement (Day 0) (Figure 2D). Postpartum females were moved together with their litter, and pups remained in the cage throughout the testing period. For postpartum females, transfer to the TPT cage occurred between postpartum day (PPD) 0 and 4 so that all measurements were completed within the early postpartum period (PPD3–7).

Each TPT session began at the onset of the light phase by providing a standardized amount of fresh bedding in the testing cage. Mice were then left undisturbed for 24 h. Plugging behavior was quantified by measuring the mass of bedding packed into the tube openings at the end of this period. For experiments involving repeated daily measurements, this 24-h measurement cycle was repeated across consecutive days without transferring mice to a different cage or returning them to group housing (Figure 2D).

### 2.4 Intruder exposure experiment

To examine whether plugging behavior is modulated by direct threat experience, a male intruder was introduced after baseline TPT sessions. Plugging behavior was first measured for two consecutive baseline sessions (Day 1 and Day 2) using the procedure described above.

Following the second baseline measurement, a male intruder was introduced into one of the tube openings. To ensure that the dam recognized the intruder within the tube, the intruder’s tail was gently held until she approached and made contact with the intruder through the tube opening. After this brief interaction, the intruder was removed. Plugging behavior after intruder exposure was quantified using the same bedding-mass measurement described for the TPT.

### 2.5 Quantification of plugging behavior

Plugging behavior was quantified by measuring the mass of bedding packed inside the openings of the two cage attached tubes. After each 24 h TPT session, the outer caps of the tubes were unscrewed, and bedding located within the inner opening of each tube was carefully removed using a small curved metal spatula. The collected bedding was transferred into pre weighed containers and measured using an electronic balance. Each tube was measured separately, and the two values were summed to obtain the plugging intensity for each mouse.

Only bedding clearly located inside the tube opening was considered plugged material. Bedding outside the opening or bedding not entering the inner opening was not included. For multi day experiments, all plugged bedding was completely removed after each measurement to ensure that each 24 h session began with an empty tube. The sole quantitative measure used in all analyses was the total packed mass expressed in grams.

### 2.6 Statistical analysis

All statistical analyses were performed using GraphPad Prism 9 (GraphPad Software, CA, USA). Normality was assessed using the Shapiro–Wilk test, which indicated that the data were not normally distributed. Therefore, non-parametric tests were used, including the Kruskal–Wallis test followed by Dunn’s multiple comparisons test and the Wilcoxon signed-rank test. To assess individual behavioral repeatability, correlations between plugging levels across days were analyzed using Spearman’s rank correlation coefficient. All tests were two-tailed, and statistical significance was set at *p* < 0.05.

## 3 Results

### 3.1 Sex and reproductive state differences in tube-plugging behavior

**Figure 3:**
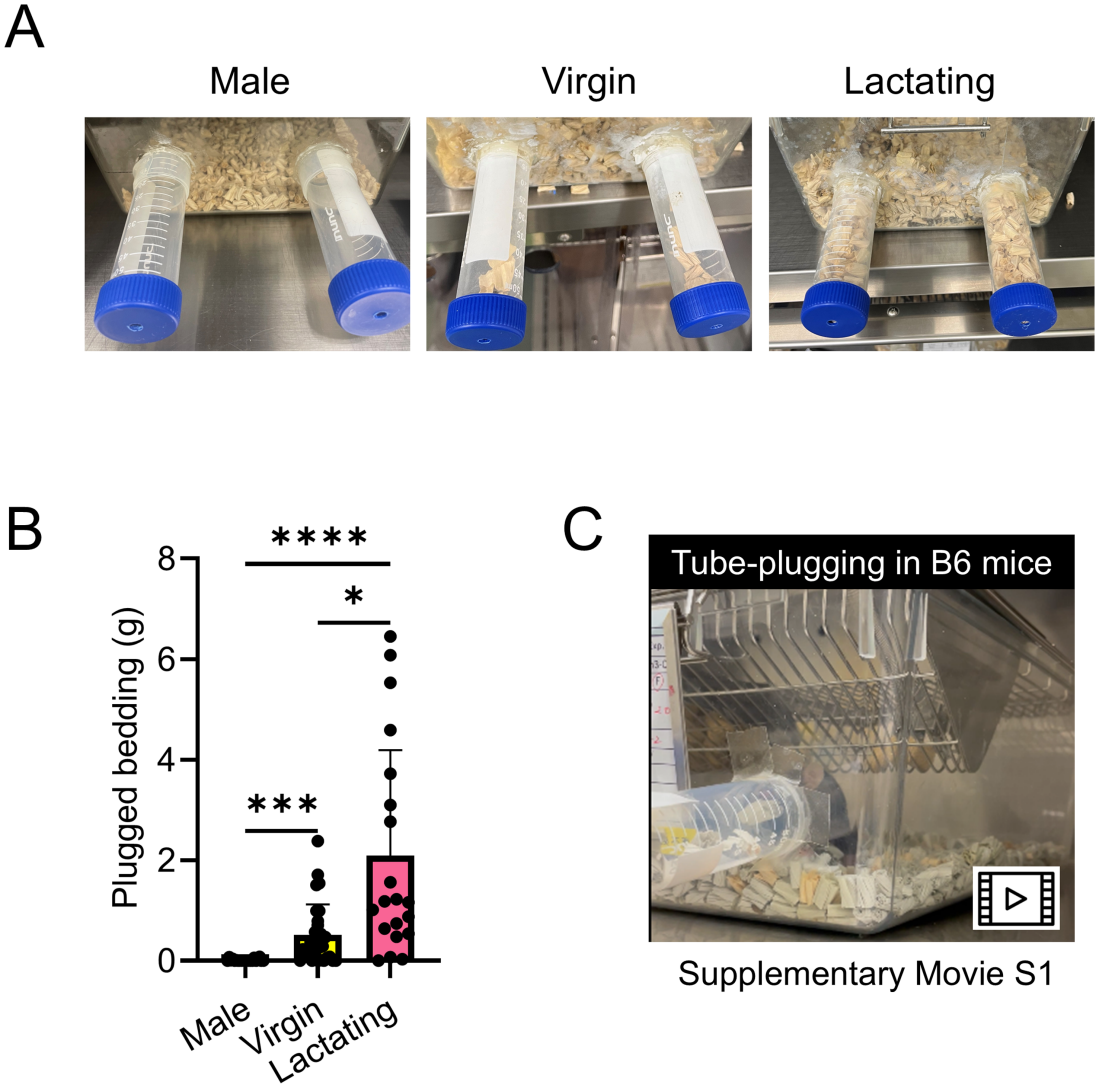
Sex and reproductive state differences in tube-plugging behavior. **(A)** Representative examples of tube-plugging behavior in males, virgin females, and lactating females. Males rarely packed bedding into the tubes, whereas females—especially lactating females—plugged large amounts of bedding material into the tube opening. **(B)** Amount of plugged bedding (g) averaged across three consecutive measurement days. Data are shown as mean ± SD. Tube plugging differed significantly among groups (Kruskal–Wallis test, *p* < 0.0001), with postpartum (lactating) females exhibiting higher levels of plugging than both males and virgin females (Dunn’s multiple comparisons test: male vs. virgin, ***p < 0.001; male vs. lactating, ****p < 0.0001; virgin vs. lactating, *p < 0.05). Each dot represents an individual animal. **(C)** Representative example of tube-sealing behavior in a C57BL/6 (B6) mouse. See Supplementary Movie S1 for the corresponding behavior.

Representative images of tube-plugging behavior in male, virgin female, and lactating female mice are shown in Figure 3A. Males rarely showed measurable plugging, whereas virgin females exhibited intermediate levels and lactating females showed more robust plugging. Because the data were not normally distributed, non-parametric tests were applied. Quantification of tube plugging revealed significant differences across sex and reproductive state (Figure 3B, mean ± SD: male, 0.013 ± 0.026; virgin female, 0.51 ± 0.61; lactating female, 2.1 ± 2.1; medians: male, 0; virgin, 0.29; lactating, 1.17; Kruskal–Wallis test, *p* < 0.0001; Figure 3B). *Post hoc* comparisons using Dunn’s test showed that lactating females exhibited greater plugging than both males (*p* < 0.0001) and virgin females (*p* = 0.002), and that virgin females also exhibited greater plugging than males (*p* = 0.0007). A representative example of tube-plugging behavior in lactating female is shown in Supplementary Movie S1 (Figure 3C). All lactating females constructed identifiable nests and gathered pups within the nest area regardless of plugging behavior (data not shown).

In ICR mice, plugging behavior was also observed in lactating females (Supplementary Figure S1). In lactating ICR females, large amounts of bedding accumulated not only within the tube openings but also across the anterior cage wall (Supplementary Figure S1A, Lactating #1) and on the cage lid (Supplementary Figure S1A, Lactating #2). Examples of mice actively packing bedding into upper cage corners in both ICR and B6 strains are shown in Supplementary Figure S1B and Supplementary Movie S2. Quantitative analysis revealed that lactating ICR females exhibited greater plugged mass than both virgin females and males, whereas no significant difference was detected between virgin females and males (Supplementary Figure S1C, mean ± SD: male, 0.022 ± 0.025; virgin female, 0.005 ± 0.014; lactating female, 1.14 ± 1.70; median: male 0.01; virgin female, 0.00; lactating female, 0.44; Kruskal–Wallis, *p* = 0.0001; Dunn *post hoc* test: male vs. virgin female, *p* = 0.82; Male vs. lactating female, *p* = 0.006; Virgin vs. lactating females, *p* = 0.0002). The amount of bedding plugged into the tubes by lactating ICR females did not significantly differ from that of lactating B6 females (Supplementary Figure S1C, Mann–Whitney, *p* = 0.24), indicating that the lactation-dependent increase in plugging behavior was observed in both strains.

**Figure 4:**
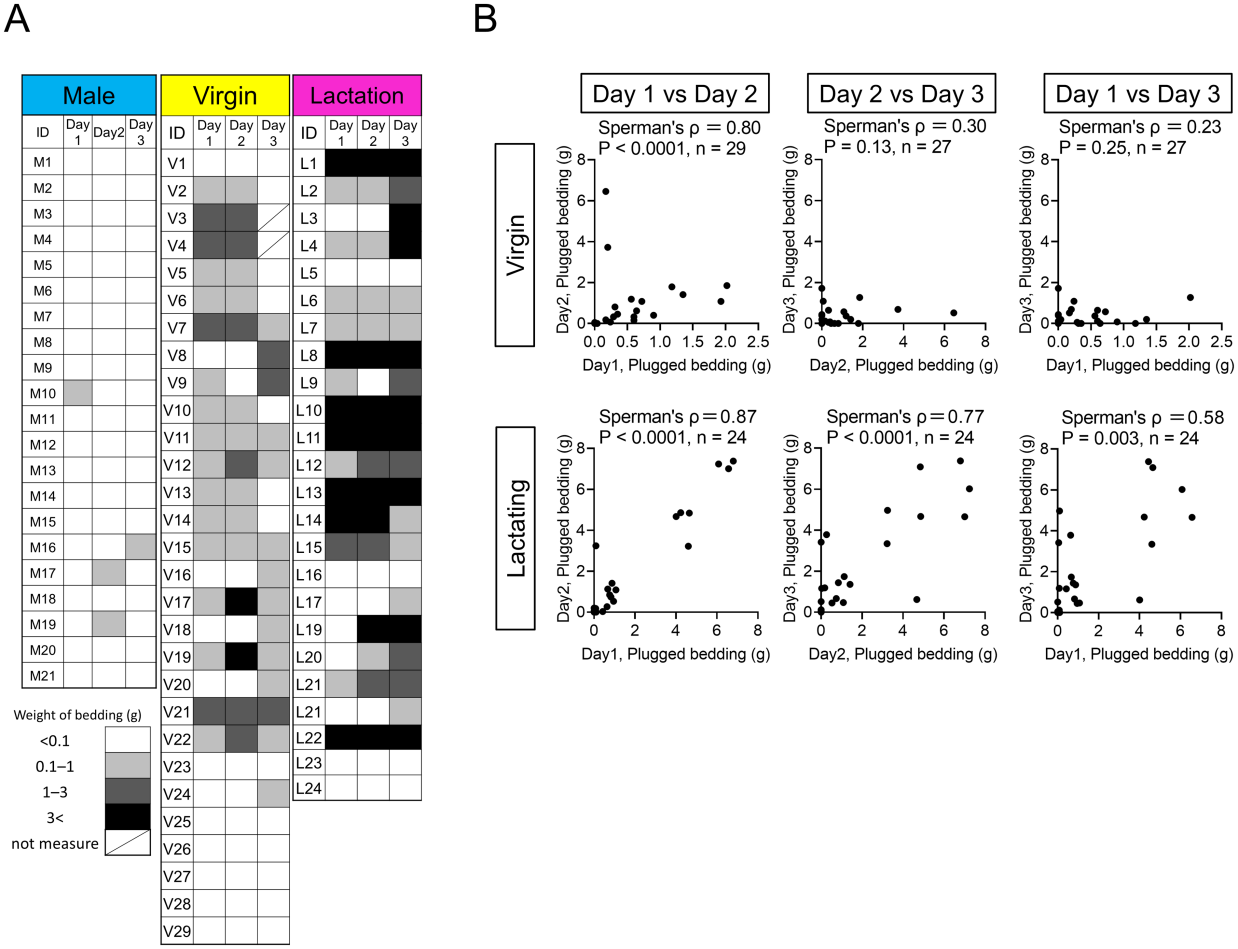
Inter-individual variability and temporal stability of tube plugging behavior. **(A)** Individual patterns of tube-sealing intensity across repeated daily sessions. Each row represents one animal and columns indicate sealing intensity for Day 1–Day 3. Shading indicates the weight of packed bedding (g), with darker shading representing greater amounts. Diagonal hatching indicates sessions that were not measured. **(B)** Correlation of tube-plugging behavior across days in virgin and lactating females. Scatter plots show pairwise comparisons of plugging amounts between Day 1 and Day 2, Day 2 and Day 3, and Day 1 and Day 3. Each dot represents an individual animal. Spearman’s rank correlation coefficients (*ρ*) and corresponding *p*-values are indicated in each panel. Lactating females exhibited higher temporal stability compared to virgin females.

A grayscale matrix representation of plugged mass across repeated sessions revealed relatively large inter individual differences within groups (Figure 4A). Individuals that inserted measurable bedding on one session tended to do so across subsequent sessions, whereas individuals that did not plug rarely initiated plugging on later days. To assess the repeatability of tube-plugging behavior, correlations between Day 1 and Day 2, Day 2 and Day3, and Day 1 and Day 3 were analyzed in virgin and lactating females. In virgin females, plugging levels were positively correlated between Day 1 and Day 2 (Figure 4B, Spearman’s *ρ* = 0.80, *p* < 0.0001, *n* = 29), whereas correlations between the other time points were not significant (Figure 4B, Day 2 vs. Day3: Spearman’s *ρ* = 0.30, *p* = 0.13, *n* = 27; Day 1 vs. Day3: Spearman’s *ρ* = 0.23, *p* = 0.25, *n* = 27). In contrast, in lactating females, plugging levels were positively correlated across all time points (Figure 4B, Day 1 vs. Day 2: Spearman’s *ρ* = 0.87, *p* < 0.0001, *n* = 24; Day 2 vs. Day 3: Spearman’s *ρ* = 0.77, *p* < 0.0001, *n* = 24; Day 1 vs. Day 3: Spearman’s *ρ* = 0.58, *p* = 0.003, *n* = 24). These results suggest that the temporal stability of tube-plugging behavior differs between reproductive states, with more consistent patterns observed in lactating females than in virgin females.

### 3.2 Intruder exposure selectively enhanced tube-plugging behavior in postpartum females

**Figure 5:**
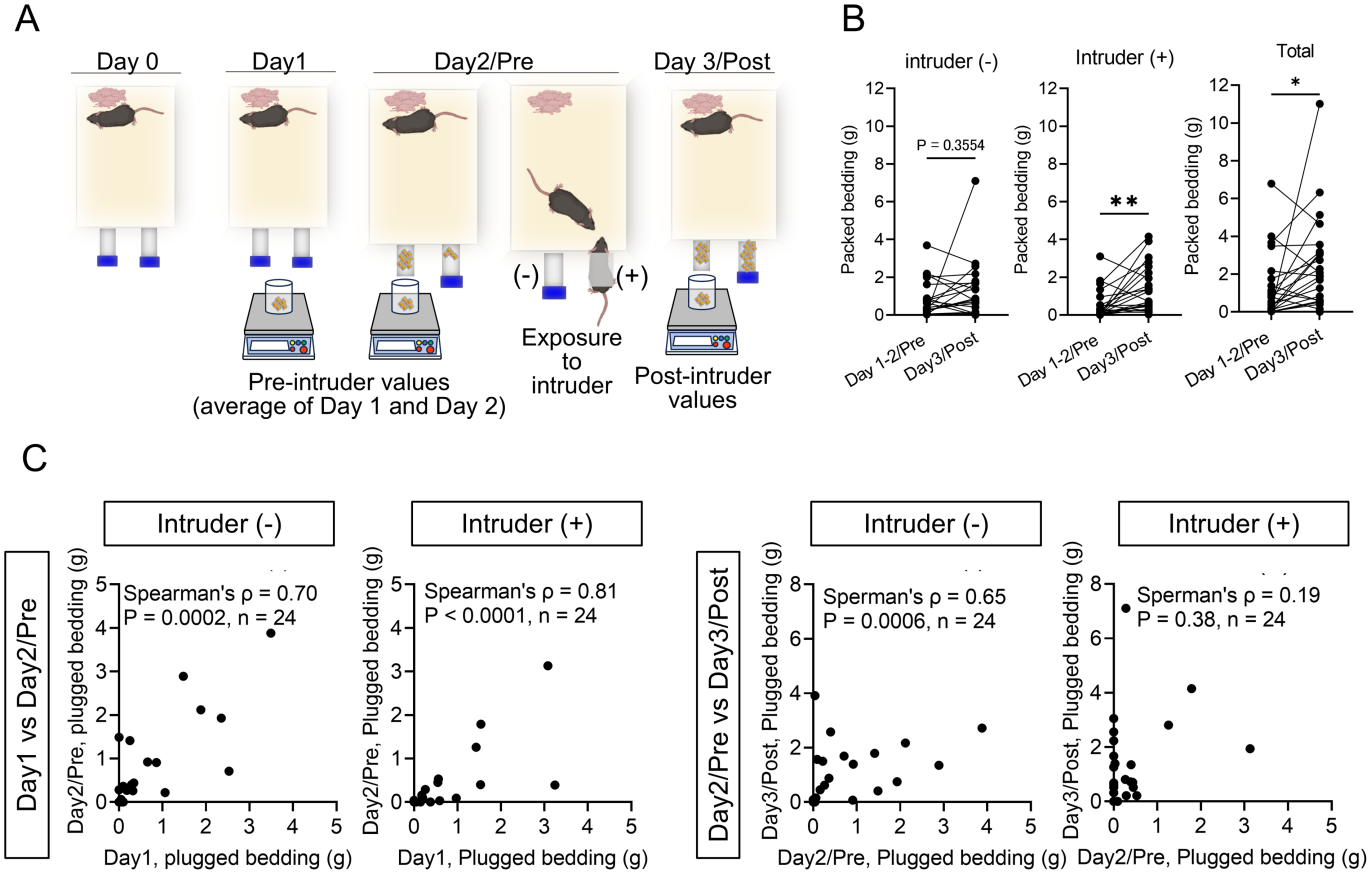
Intruder-induced modulation of tube plugging behavior. **(A)** Experimental timeline for the intruder assay. Postpartum females were transferred to the TPT cage on Day 0. Tube-plugging behavior was quantified on Day 1 and Day 2 to obtain pre-intruder values (mean of Day 1 and Day 2). On Day 2, a male intruder was introduced through one of the tubes, allowing brief direct contact before release into the cage. Plugging behavior was quantified again on Day 3 to obtain post-intruder values. **(B)** Changes in tube-plugging behavior following intruder exposure. Paired comparisons of plugging amounts between pre-intruder (Day 1–2 mean) and post-intruder (Day 3) conditions are shown for the intruder-exposed tube (Intruder +), the non-exposed tube (Intruder −), and the total amount. Each line represents an individual animal. Statistical significance was assessed using the Wilcoxon signed-rank test, and *p*-values are indicated in each panel (*p < 0.05, **p < 0.01). **(C)** Correlation of tube-plugging behavior before and after intruder exposure. Left panels show correlations between Day 1 and Day 2 (pre-intruder) values, and right panels show correlations between pre-intruder (Day 2) and post-intruder (Day 3) values. Data are shown separately for the intruder-exposed tube (Intruder +) and the non-exposed tube (Intruder −). Each dot represents an individual animal. Spearman’s rank correlation coefficients (*ρ*), *p*-values, and sample sizes are indicated in each panel.

We next examined how intruder exposure affected both the intensity and the temporal stability of tube-plugging behavior in lactating females (Figure 5). Following two baseline sessions (Day 1–2), a male intruder was introduced through one of the tubes on Day 2, and plugging behavior was quantified again on Day 3 (Figure 5A). Plugging intensity on the tube not exposed to the intruder showed no significant change between pre-intruder value (mean value of day 1 and day 2) and post-intruder value on day 3 (Figure 5B, Intruder (−), mean ± SD: Pre, 0.73 ± 0.95; Post, 1.12 ± 1.54; medians: Pre, 0.32; Post, 0.73; median: Pre, 0.31; Post, 0.72; Wilcoxon signed-rank test, *p* = 0.36). In contrast, plugging intensity on the intruder-exposed tube increased significantly after intruder exposure (Figure 5B, Intruder (+), mean ± SD: Pre, 0.49 ± 0.77; Post, 1.28 ± 1.25; medians: Pre, 0.16; Post, 0.70; Wilcoxon signed-rank test, *p* = 0.0043). Total plugging across both tubes was also significantly elevated following intruder exposure (mean ± SD: Pre, 1.21 ± 1.70; Post, 2.40 ± 2.50; medians: Pre. 0.47; Post, 1.89; Wilcoxon signed-rank test, *p* = 0.0179).

To assess the effect of intruder exposure on the stability of tube-plugging behavior, correlations across days were analyzed separately for the intruder (−) and intruder (+) tubes (Figure 5C). Prior to intruder exposure (Day1 vs. Day2), plugging levels were positively correlated in both the intruder (−) tube (Spearman’s *ρ* = 0.70, *p* = 0.0002, *n* = 24) and the intruder (+) tube (*ρ* = 0.81, *p* < 0.0001, *n* = 24). Following intruder exposure (Day2 vs. Day3), the positive correlation was maintained in the intruder (−) tube (Spearman’s *ρ* = 0.65 *p* = 0.0006, *n* = 24), whereas no significant correlation was observed in the intruder (+) tube (Spearman’s *ρ* = 0.19, *p* = 0.29, *n* = 24). These results indicate that intruder exposure selectively disrupts the stability of tube-plugging behavior in the intruder-exposed tube.

## 4 Discussion

In the present study, we established the TPT as a simple assay that enables quantification of bedding-plugging behavior in mice. Tube plugging showed a strong sex bias, being rarely observed in males and most robustly expressed in postpartum females. Although virgin females engaged in plugging, postpartum females exhibited greater plugging intensity. Notably, tube-plugging behavior exhibited substantial inter-individual variability across animals. Individual differences in tube-plugging behavior were more stable across days in lactating females than in virgin females. In addition, intruder exposure selectively increased plugging at the tube through which the intruder was introduced, indicating that plugging intensity is sensitive to recent threat experience. This temporal stability was maintained in the non-exposed tube but not in the intruder-exposed tube, where plugging tended to increase following intruder exposure. This suggests that recent intruder experience alters both the magnitude and consistency of plugging behavior in a context-dependent manner. Together, these findings establish tube plugging as a measurable behavioral pattern that is enhanced during the postpartum period and dynamically modulated by recent intruder exposure.

The present findings raise the question of how tube-plugging behavior relates to other bedding-based behaviors, such as nest building, that have been extensively studied in laboratory rodents (Hess et al., 2008). Many species-typical behaviors, including nest building, digging, and burrowing (Deacon, 2006a,b; Pond et al., 2021), involve the use or displacement of material to generate or maintain usable space for functions such as foraging, refuge, and offspring care. In nest building, bedding material is organized at the site where the animal or its offspring are located, forming a structure that can be occupied. In contrast, entrance-sealing behavior observed in species such as rats and rabbits (Calhoun, 1963; Rodríguez-Martínez et al., 2014) involves the closure of an opening, thereby restricting access to a specific location. The tube-plugging behavior observed in the present study shares this latter characteristic, in that bedding material is accumulated at the tube opening, spatially separated from the location of the animal or its pups, resulting in the occlusion of an opening rather than the creation of usable space. Importantly, both plugging and non-plugging lactating females constructed clearly identifiable nests and gathered pups within the nest area, indicating that tube plugging occurs alongside intact nest-building behavior and does not reflect a disruption of it. Rather than being an extension of nest building, tube plugging appears to represent a functionally distinct form of bedding-based environmental modification.

Tube plugging exhibited substantial inter-individual variability in both virgin and lactating females, with a degree of temporal repeatability across days. Although rarely observed in males, occasional individuals showed low levels of plugging. The sources of this variability remain unclear, including whether it reflects innate circuit differences or experience-dependent factors. Notably, entrance-sealing behavior in rodents has been reported to vary with social rank and dominance status, with lower-ranking individuals showing a greater tendency to seal (Calhoun, 1963), suggesting that internal state and social context may contribute to this variability. Although entrance-sealing behavior in species such as rats and rabbits has been interpreted as a form of defensive behavior, it remains unclear whether tube plugging in the present assay can be directly categorized in the same way. One possible explanation is that the tube openings used in the TPT create narrow, tunnel-like structures whose interior cannot be directly inspected, thereby introducing spatial uncertainty. Unlike the open front of standard cages, the tubes create confined spaces whose distal ends are not visible to the animal. In rodents, investigation of holes or tunnels—such as in the hole-board test—is now considered to reflect not merely exploration, but also risk assessment, emotional reactivity, and active coping strategies (Pisula et al., 2021). In this context, the tube openings used in the TPT may represent spatially uncertain structures that evoke active responses in lactating females.

A limitation of the present study is that the mechanisms underlying tube-plugging behavior were not directly examined. In particular, factors such as maternal age, reproductive state, associated hormonal changes, and environmental context were not systematically examined, and their respective contributions remain to be clarified. Future studies will be required to address these factors and to determine the extent to which tube plugging reflects responses to spatial uncertainty, for example through pharmacological manipulation using anxiolytic agents. In addition, the substantial inter-individual variability observed in this behavior warrants further investigation into its biological and environmental determinants.

## Data Availability

The raw data supporting the conclusions of this article will be made available by the authors, without undue reservation.
