# Supplementary figures and images for: The tube-plugging test: a simple assay that reveals offspring-centered defensive behavior in postpartum mice
Source: Front Behav Neurosci. 2026 May 26;20:1767805. doi: 10.3389/fnbeh.2026.1767805 (PMC13246676; doi:10.3389/fnbeh.2026.1767805)

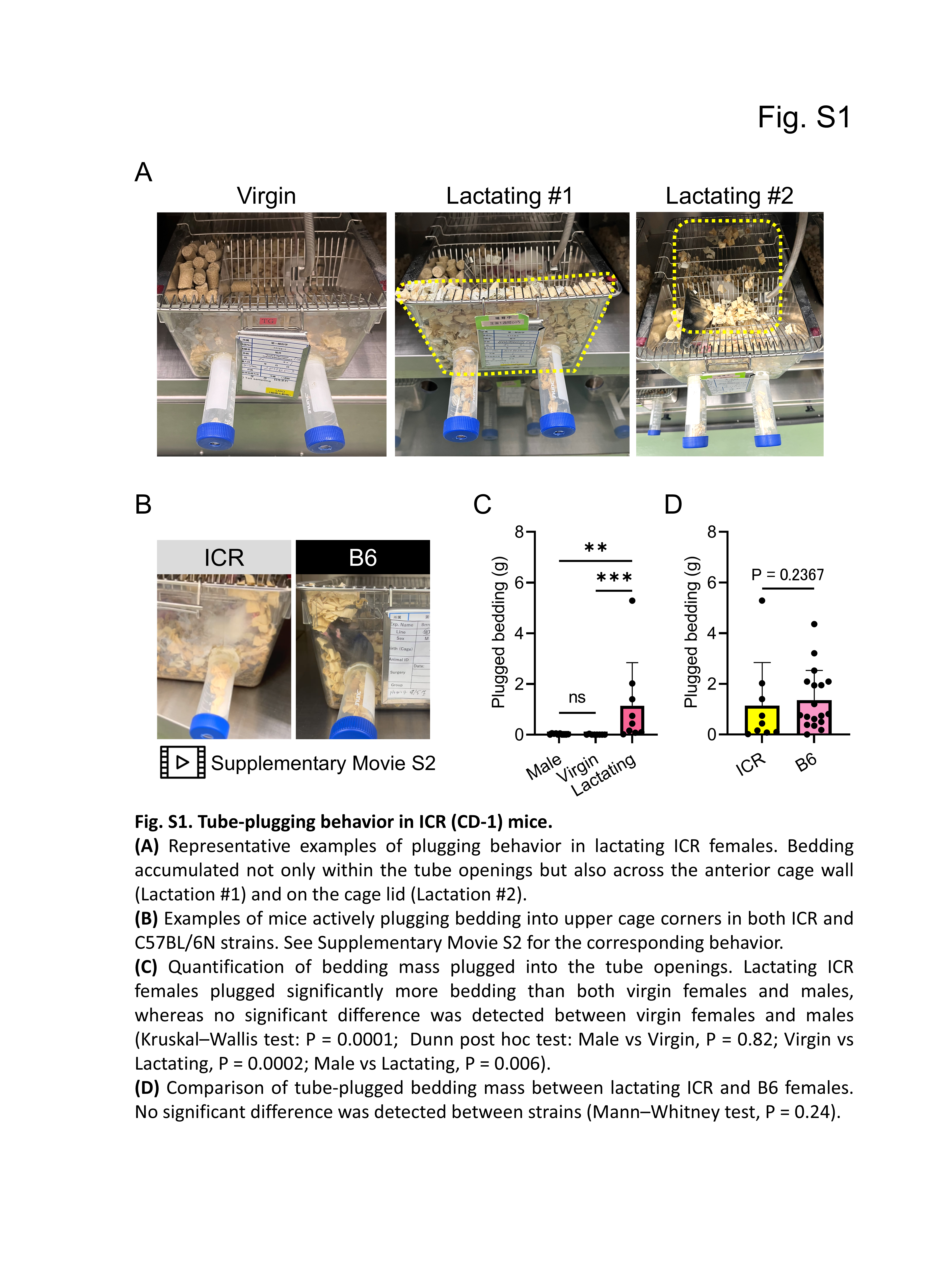

Supplement: Supplementary file 1 [file Image_1.TIF]
